# Treatment-Seeking Delay Among Hispanic and Non-Hispanic Women with Acute Myocardial Infarction

**DOI:** 10.1089/heq.2018.0046

**Published:** 2019-06-24

**Authors:** Amy Pate, Bonnie A. Leeman-Castillo, Mori J. Krantz

**Affiliations:** ^1^ Department of Family Medicine, University of Colorado—Anschutz Medical Campus, Aurora, Colorado.; ^2^ Colorado Prevention Center, Aurora, Colorado.; ^3^ Cardiology Division, Denver Health Medical Center, Denver, Colorado.

**Keywords:** Hispanic women, acute myocardial infarction, treatment-seeking behavior, prehospital delay, ethnic variables

## Abstract

**Purpose:** Women and minorities with acute myocardial infarction (AMI) often fail to recognize prodromal symptoms leading to delays in care. The objective of this study was to conduct a mixed method assessment of the impact of ethnicity on symptom description, recognition, and treatment-seeking behavior in Hispanic and non-Hispanic women before hospitalization for AMI.

**Methods:** We explored differences in symptomatology, treatment-seeking behavior, and delay patterns among a convenience sample of 43 women diagnosed with AMI (17 Hispanic women, 26 non-Hispanic women) in seven rural and urban Colorado hospitals. We performed in-depth interviews to establish patterns (typologies) of treatment-seeking behaviors. Chart abstraction provided delay times as a function of ethnicity.

**Results:** Most (28/43) women reported prodromal symptoms in the weeks before their index AMI. Overall, fewer Hispanic women presented within 24 h of symptom onset (3/18, 17% vs. 15/18, 83%, *p*<0.01). A typology of treatment-seeking behavior emerged: women who (1) recognized symptoms and promptly sought care; (2) did not recognize symptoms, yet promptly sought care; (3) recognized symptoms and promptly sought care, but providers misconstrued symptoms as noncardiac; and (4) misinterpreted symptoms due to an underlying chronic disease.

**Conclusion:** Women and primary care providers often underappreciate prodromal AMI symptoms. Hispanic women are more likely to misinterpret ischemic symptoms and delay care, suggesting a need for tailored patient and provider education.

## Introduction

Acute myocardial infarction (AMI) is the leading cause of death for American women regardless of ethnicity.^1–3^ Although women have their first AMI an average of 10 years later than men, they are more likely to have comorbidities that engender atypical symptoms.^1,2^ Women experience greater morbidity and higher rates of mortality after AMI compared with men with similar baseline characteristics; this disparity persists from hospitalization through 1 year after discharge.^2,4–11^ Mortality associated with AMI is also higher for minorities. Despite controlling for patient and hospital characteristics, the reasons for this disparity remain poorly defined.^2^

Women and ethnic minorities experiencing AMI symptoms are more likely to delay seeking care and have longer mean delay times than men and white individuals. ^1,2,9,12–16^ Although this is a well-established finding, there is a paucity of scientific literature focused on the contextual variables^4,12–14,17^ associated with women's experiences seeking treatment; furthermore, less is known about the aforementioned variables affecting Hispanic individuals.

Women who delay seeking care often fail to recognize the importance of prodromal AMI symptoms.^5,12–14^ Little is known about the variation attributable to ethnicity in women's perceived symptomatology and how this impacts emergency treatment-seeking behavior. Informed by the Health Belief Model and other behavioral science theories,^12,18–21^ the objective of this study was to conduct a mixed method assessment of the impact of ethnicity on symptom description, recognition, and treatment-seeking behavior in Hispanic and non-Hispanic women before hospital admission for AMI.

## Methods

We recruited adult women with AMI from seven urban and rural hospitals in Colorado, including counties with substantial Hispanic populations (Denver, Greeley, and Pueblo).^22^ The mixed method design employed semistructured interviews with subsequent medical record abstraction; subjects were given a ten-dollar grocery store gift certificate as incentive for participation. Written informed consent was obtained from all participants. The Colorado Multiple Institutional Review Board approved the study.

### Recruitment

Purposeful sampling relied on inpatient nursing staff to identify potential participants. Eligibility was limited to clinically stable, English- or Spanish-speaking women 18 years of age or older admitted with an AMI diagnosis as defined by the Third Universal Definition criteria.^23^ All subjects had a primary International Classification of Diseases, Ninth Revision, Clinical Modification (ICD-9-CM) admission diagnosis of AMI and required elevation of cardiac biomarkers. Exclusion criteria included male gender, presence of delirium or dementia, and unwillingness or inability to provide written informed consent. Participants were enrolled sequentially until no new themes emerged from the interview process.

### Interviews

Participants completed one face-to-face interview while hospitalized and a follow-up interview ∼2 weeks after discharge with a behavioral scientist (B.A.L.-C.). Interview topics included women's attitudes, beliefs, and experiences related to AMI symptoms; social support networks within which women discussed their symptoms; and women's experiences accessing care. Delay time was defined as the time between self-reported symptom onset and hospital arrival. In follow-up interviews, women clarified and expanded on their experiences, enhanced identification of prodromal symptoms, and validated transcribed data. For Spanish-speaking participants, we trained two language-certified bilingual Hispanic women to conduct interviews and assist with transcription to augment the non-native Spanish language skills of B.A.L.-C. The behavioral scientist participated in all interviews, including those conducted in Spanish to assure content consistency and collect nonverbal data. All interviews were audiorecorded, transcribed verbatim, and translated into English.

### Chart review

We reviewed hospital records and laboratory data to compare official reports with interview findings. Record review assessed narrative descriptions documented by emergency response teams and emergency department staff. The data collection instrument captured demographics, family history, ambulance and emergency department reports, cardiac biomarkers, medical history summary, and discharge information.

### Analyses

The behavioral scientist analyzed all interviews using an inductive constant comparative approach^17^ to identify and code themes, reveal participants' explanatory models, condense transcripts into vignettes, and derive a typology.^12,24,25^ Using fictitious names for participants, we created Patient Career Timelines (PCTs) as an analytic tool to temporally visualize and assess individual and group treatment-seeking behavior. PCTs are visual maps that capture symptoms, levels of care, communication, and participants' thoughts and perceptions across a specified time period.

To derive our typology, 43 timelines were categorized by (1) presentation within 24 h, (2) patient's recognition of potential AMI symptoms, and (3) actions taken in response to these symptoms. The vignettes, PCTs, and chart reviews permitted triangulation^24^ of the data to verify how the AMI event unfolded. Triangulation augmented information and permitted discovery of inconsistencies between the medical charts and vignettes. Peer auditors verified the research process and findings. Discrepancies were pursued through additional data review and discussed until agreement was reached among the investigators.

Categorical variables were compared through chi-square analysis or Fisher's exact test, as appropriate; continuous variables were compared using a two-sample *t*-test. First positive troponin-I levels and total delay time were not normally distributed and thus log-transformed prior linear regression. The principal quantitative outcome was a comparison of the proportion of participants with delayed presentation (>24 h) by ethnicity. Statistical analyses were performed using SAS (version 9.0; SAS Institute, Cary, NC).

## Results

A total of 58 women were identified as potential participants. Six eligible women declined to participate and nine were not enrolled due to brief hospitalizations. Of the 43 women who consented to participate, 41 completed both interviews (1 expired and the other was lost to follow-up). All subjects were included in analyses.

Characteristics of the study sample are depicted in Table 1. Hispanic women represented 40% of the total sample (*n*=17). Sixteen of 17 (94%) Hispanic women self-reported being of Mexican American heritage. A higher percentage of Hispanic women had less than a 12th grade education compared with non-Hispanic women (88% vs. 27%, *p*<0.001). Five of the 17 Hispanic women preferred speaking Spanish for their interviews. A total of 23 women (53%) were recruited from rural hospitals. All women underwent percutaneous coronary intervention. Most women included in the overall sample (60%) had one or more underlying comorbidity, including: diabetes (*n*=6), arthritis (*n*=7), established cardiovascular disease (*n*=5), prior AMI (*n*=4), or chronic obstructive pulmonary disease (*n*=4).

**Table 1.** Patient Characteristics by Ethnicity

	**Total sample (*n*=43)**	**Hispanic women (*n*=17)**	**Non-Hispanic women**^a^**(*n*=26)**	***p***
Age (years)				
Mean	62±11.9	62±11.4	62±12.5	0.983
Range	39–89	42–83	39–89	
Highest level of education				<0.001^a^
<12th Grade	22 (51%)	15 (88%)	7 (27%)	
≥12th Grade	21 (49%)	2 (12%)	19 (73%)	
Health insurance				0.481
Insured	33 (77%)	12 (71%)	21 (81%)	
Uninsured	10 (23%)	5 (29%)	5 (19%)	
Marital status				0.911
Single	4 (9%)	2 (12%)	2 (8%)	
Married	18 (42%)	6 (35%)	12 (46%)	
Widowed	10 (23%)	4 (24%)	6 (23%)	
Divorced/separated	11 (26%)	5 (29%)	6 (23%)	
Lives alone				0.722
Yes	14 (33%)	5 (29%)	9 (35%)	
No	29 (67%)	12 (71%)	17 (65%)	
Location				0.692
Rural (population <25,000)	8 (19%)	4 (24%)	4 (15%)	
Urban (population >25,000)	11 (26%)	3 (18%)	8 (31%)	
Mixed^b^	24 (56%)	10 (59%)	14 (54%)	
Co-morbidity influenced patient's assessment of symptoms^c^				0.646
Yes	26 (60%)	11 (65%)	15 (58%)	
No	17 (40%)	6 (35%)	11 (42%)	

The non-Hispanic sample included 25 white women and 1 black woman.

^a^ Denotes statistical significance at *p*<0.05.

^b^ A mixed community is a small town located near a larger community and is assigned the zip code affiliated with the larger town. Fisher's exact test calculated by comparing Hispanic women versus non-Hispanic women and combined location categories: rural versus urban+mixed.

^c^ Diabetes, chronic obstructive pulmonary disease, osteoporosis, arthritis, prior AMI or other cardiovascular disease, gastrointestinal disorders.

AMI, acute myocardial infarction.

### Characteristics of participants' symptoms

**FIG. 1.**
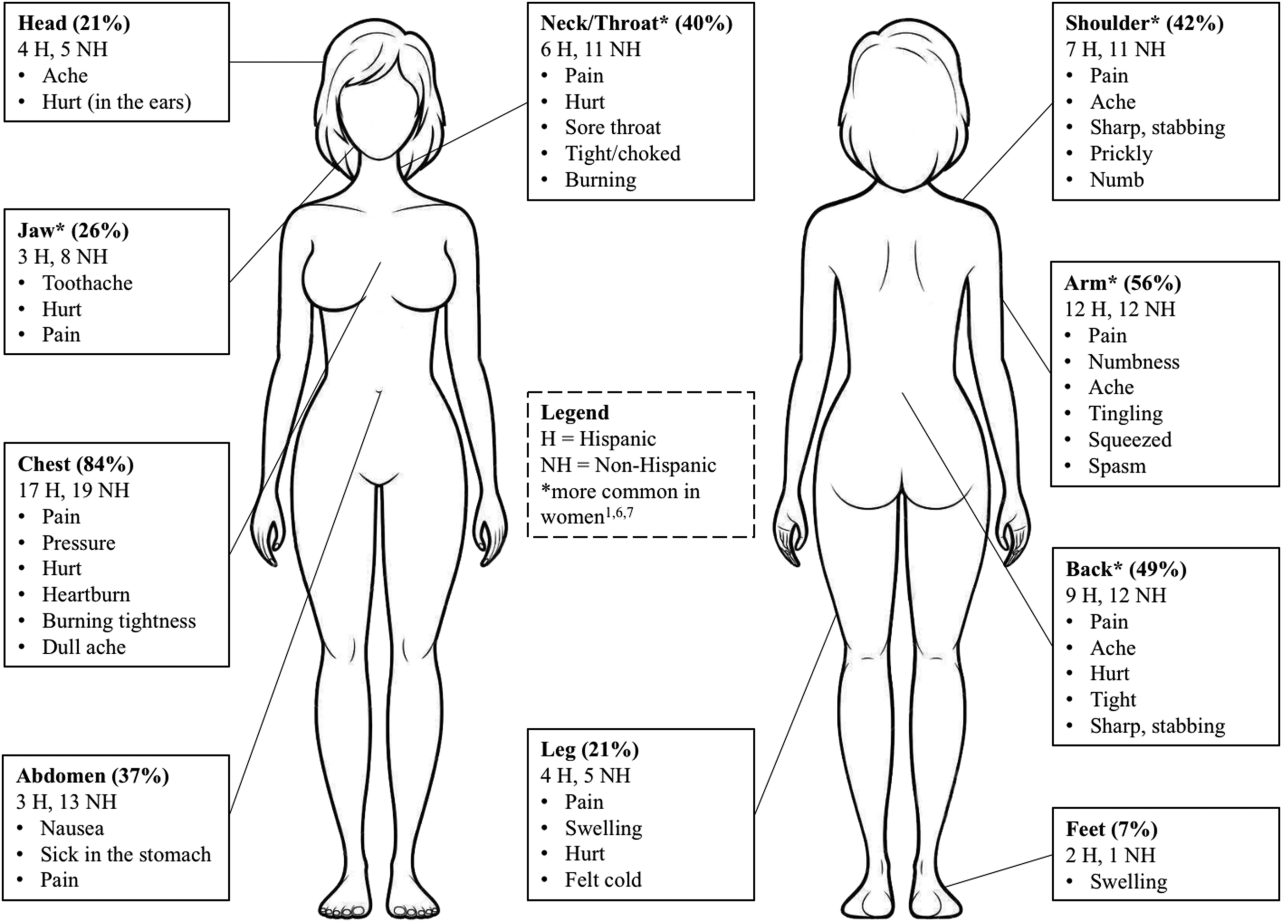
Location and quality of symptoms.

Figure 1 illustrates participants' symptoms by location and the most common words used to describe the quality of their symptoms. Many participants, particularly those in whom AMI evolved over several days, identified multiple locations for their symptoms. Only 35% of women described their symptoms as severe or intolerable. Most women described symptoms as mild (tolerable) or moderate. More than 70% of women explained that they delayed seeking care because their symptoms were not severe and therefore did not meet their expectations for an AMI.

Sixty-five percent (*n*=28) of women described intermittent symptoms that were not taken seriously. As symptoms were incongruent with participants' expectations for AMI, this prompted a “wait and see” approach to seeking care. Most often, symptoms began as intermittent and evolved into constant on the day of hospitalization.

Approximately 84% (*n*=36) of women reported chest pain. However, symptoms atypical for coronary ischemia featured prominently across many symptom profiles. Participants often assessed their symptoms' origin as noncardiac. Fifty-three percent of all women reported dyspnea and 42% reported symptoms that disrupted sleep. More than 25% of participants mentioned “tiredness,” fatigue, sweating, malaise, dizziness, or feeling cold. These associated symptoms were often incompatible with women's expectations of an AMI.

### Delay time

Of the 18 women who presented promptly (<24 h), 3 (17%) were Hispanic and 15 (83%) were non-Hispanic (*p*<0.01). There was no evidence of an association between delay time and troponin-I levels. The mean first positive troponin-I level measured was numerically greater but not statistically significant (*p*=0.43) among Hispanic women (16 ng/dL, range 0.3–97.5) compared with non-Hispanic women (9 ng/dL, range 0.3–49.9).

### Themes

A number of themes emerged from vignette analysis and were grouped into core categories. Themes emerging from vignette analysis were grouped into five core categories associated with recognition and response to symptoms (Table 2). These include: communicating with family and health care professionals; treating the symptoms; appraising the symptoms; being a patient; and themes more commonly found among Hispanic women. Evidence of these themes can be found throughout our typology.

**Table 2.** Selected Themes Associated with Recognition and Response to Acute Myocardial Infarction Symptoms

**Themes**	**Examples**
(A) Communicating with family and health care professionals	
(1) Recent encounters impact explanatory models	“[The PA] said, “Oh no. Those symptoms aren't consistent with a heart attack and he would lean more towards the gallbladder because [I] wasn't sweating … [I] still kinda wasn't thinking it was the heart”
(2) Selectively disclosing and altering symptom descriptions for different people	To her doctor, she detailed symptoms as “little tight cramps” in her chest during the night that caused her to “catch my breath … it was like a tightness but not, not too strong … just enough to bother me to let me know it was there” but later, to her daughter, vaguely described symptoms as “something's wrong … I just don't feel good.”
(B) Treating the symptoms	
(1) Actively obtaining care	“Don't mess with the pain THERE. [You] just call 911.”
(2) Knowing the treatment—expressing certainty of the level of treatment needed	“I've got to let my doctor see me feeling this icky … I'm going to show her that something is wrong.”
	“the nitro would kick it”
	“I know we can't afford it … but … I'm getting really tired of the pain … and I need to go see somebody about it … to find out what is going on.”
(3) Self-treatment strategies	Treating symptoms until they became intolerable
	Failing to justify the need for immediate medical care because “familiar” symptoms were alleviated with self-treatment.
	Taking care of herself “to avoid bothering others”
	“Hoping the symptoms would disappear”
	“The way I was raised … you just handle whatever had to be done … I was born and raised on a farm and we worked hard … never had time to worry about anything … never went to the doctor … if you got hurt, you took care of it …”
(C) Appraising the symptoms	
(1) Acknowledging hunches and gut feelings	“just knew”
(2) Expecting different symptoms from those experienced	“just a chest pressure, not lightheaded like my first MI”
	“no chest pain, just a little bit of pressure”
	“When you have a heart attack … you have heaviness in your chest … that didn't happen to me”
(D) Being a patient	
(1) Crying wolf	“I truly believe I am having a heart attack right now. I'll bet you anything that when we get to the hospital, they're not going to take me very seriously. They usually listen to men.”
	I felt foolish coming in here. I had no real symptoms, you know … because I couldn't tell them anything specific. Nothing was overwhelmingly painful. It was just an extreme … pressure, tiredness … I thought I was making a lot of it … I really thought that at the emergency room, they'd have told me, “Who is this that came in and wasted our time and there is nothing wrong?”
	“It's ridiculous that other people go to the doctor every time [they] feel a pinch. If you go to the doctor every time you have a little minor thing, then they are gonna get so they don't pay any attention to you.”
(2) Learning the system	“I didn't want to come to the emergency room … it's a mandatory 48 hour visit for chest pain …”
	“I never like to go to the hospital because I'm always sick. My sugars are high all the time and all I do is sit in the waiting room forever and then I see a doctor. All they do is put IVs on me and send me home.”
(E) Common themes among Hispanic women	
(1) Praying (fatalism)	“I said to Him, God I am here at your will [when] you want to take it … I prayed and asked God [to make the symptoms go away]”
	“Maybe I will feel better if I go to church”
(2) Using home remedies before seeking care	Relieving heartburn with milk or juice, “it would kind of soothe it”
	Rubbing “heat lotion” (eucalyptus and aloe vera) to relieve pain
	Drinking “manzanilla” (chamomile tea) to calm down
(3) Being afraid of the doctor	“afraid of the doctors, the injections … and to be there in the hospital because … I don't like to go to the hospital”
(4) Involving the daughter	Asking the daughter about seeing a doctor
	Coming to a consensus with daughters about symptoms
(5) Being unaware of ischemic symptoms	“What are they? What do you do?”
	Never thought burning was related to the heart

IV, intravenous catheter; MI, myocardial infarction; PA, physician assistant.

### Typology of treatment-seeking behavior for AMI symptoms

A typology of treatment-seeking behavior emerged from our analysis. We generated qualitative labels as shorthand descriptors of the four types of participants identified (Table 3):

- Fast Actors: recognized AMI symptoms and promptly sought care in <24 h
- Got Luckies: did not recognize AMI symptoms yet promptly sought care in <24 h
- The Derailed: recognized symptoms as important but medical providers misconstrued symptoms as noncardiac (delay time >24 h)
- Wonder a Lots: misinterpreted symptoms as noncardiac due to an underlying chronic disease (delay time >24 h).

**Table 3.** Treatment-Seeking Typologies by Ethnicity

	**Overall (*n*=43)**	**Hispanic women (*n*=17)**	**Non-Hispanic women (*n*=26)**
Fast Actors	16	3	13
Got Luckies	2	0	2
The Derailed	7	5	2
Wonder a Lots	17	9	8

Fast Actors are active treatment seekers who experienced typical ischemic symptoms. This type represented 37% (3 Hispanic women, 13 non-Hispanic women) of the total sample.

Ms. Wilson: I don't know what's going to happen here, but I'm afraid … I'm going to do it and I did 911…I was pretty sure I was having a heart attack …

Mrs. Gallagher: It's not easing up … Ok. What do they tell you? Do not mess with any kind of pain … over there. Call 911 … Just call 911 …

Got Luckies experienced atypical symptoms and failed to recognize them as cardiac in origin. The two women in this typology got lucky because a spouse or key person acted on the participant's behalf and facilitated the decision to seek care. This type represented 5% (two non-Hispanic women) of the sample.

Mrs. Robbins: Well, I must be catching some kind of bug … I had a horrible headache all night long.” Her husband replied, “[Do] you want me to take you to the emergency room because that doesn't sound normal for you … you're not a headache person.

The Derailed women actively sought treatment for their symptoms before hospitalization. Most recognized their symptoms as important and sought care from a primary care provider or an emergency department. Although a few women thought their symptoms were “heart-related,” this type had their beliefs “derailed” or “devalued” when they sought medical care. Some reported that health care providers informed them that “nothing was wrong with their heart” and were treated for a different illness. These interactions affected how participants interpreted and acted upon symptoms that persisted after the provider visit. This type represented 19% (five Hispanic women including three who preferred speaking Spanish, two non-Hispanic women) of the sample.

Mrs. Galindo: I couldn't quite make [my doctor] understand that I was telling him how I felt because he would just cut in and tell me, “Oh, your heart is fine. It checks fine … So I just took it from there … nothing to worry about. He told me that it was arthritis … I'd say ‘Oh, God, I hope I'm not going to get a heart attack!’ … I *did* tell [my other doctor] about the tightness at night because he asked me if I had a hard time breathing … but he didn't seem to think anything about it. He just like bypassed it too.”

**FIG. 2.**
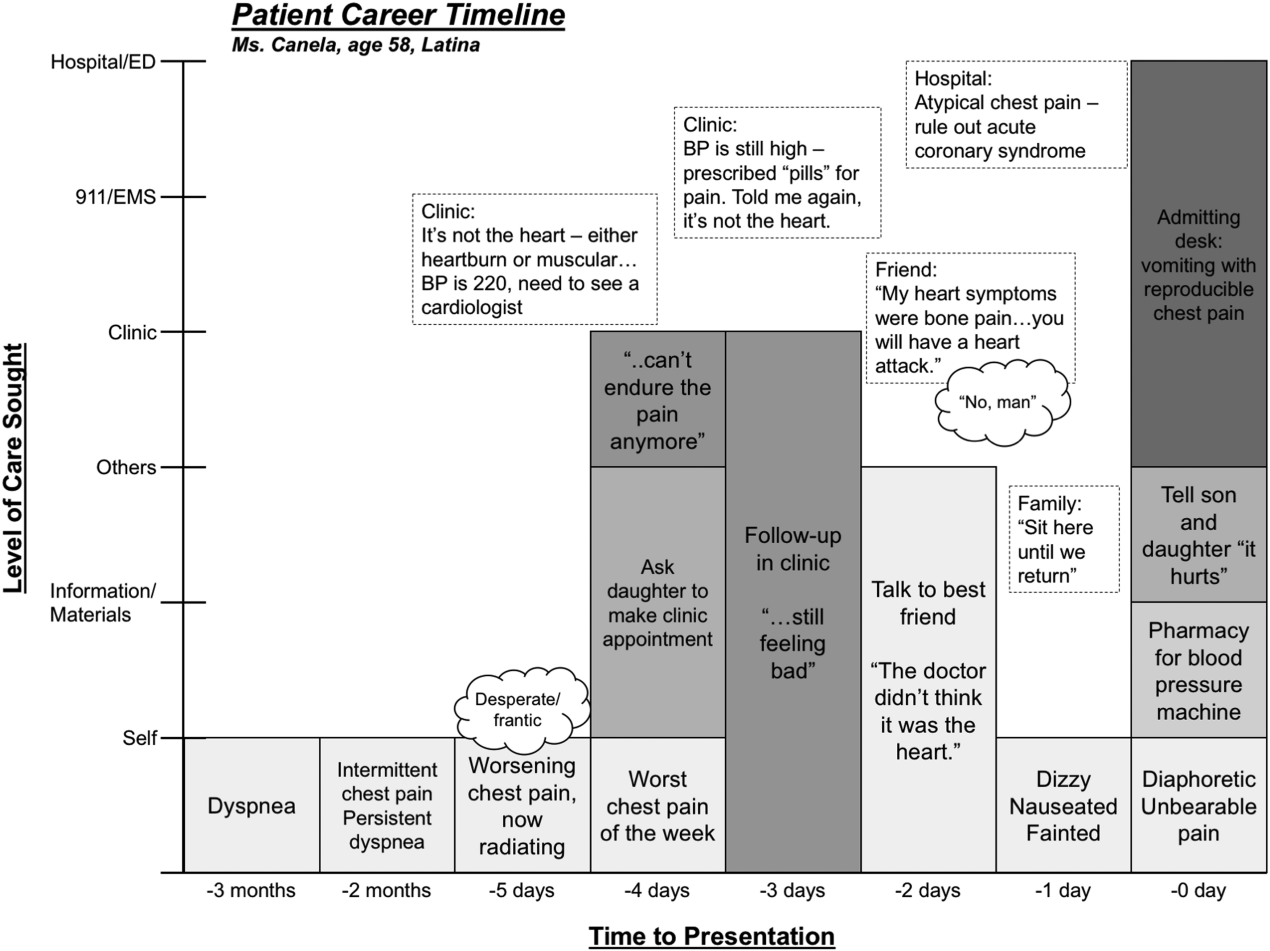
Selected PCT for “The Derailed” typology. Six stages of care are displayed on the *y*-axis: self-care, information/materials (Internet, magazines, etc.), others (family, friends, key individuals, or other nonmedical persons), clinic/private doctor (includes calls made to ask-a-nurse systems), 911/EMS, and the emergency department/hospital (highest level of seeking care). Excerpts framed by a *dashed line* represent information communicated to the patient. *Thought bubbles* represent the patient's thoughts/feelings at the time. EMS, Emergency Medical System; PCT, Patient Career Timeline.

The PCT shown in Figure 2 depicts a Spanish-speaking Hispanic woman who recognized the importance of her symptoms, described them as unbearable, sought care in the clinic twice, yet received the message that her pain was “not the heart.” This patient endured her pain for three additional days before hospital admission.

Women represented in the Wonder a Lots type expressed multiple explanations for their symptoms but were slow to seek care and focused on self-treatment. Several of these women suffered from chronic conditions such as arthritis, gastroesophageal reflux, or diabetes that were deemed bothersome but not perceived as severe. This type represented 40% (nine Hispanic women, eight non-Hispanic women) of the sample.

Mrs. Cortes: “probably catching a cold … you know how you get sore in there.” … Everybody has heartburn … didn't think it was anything serious … I *always* sweat in bed. And I'm *always* nauseated [because of my diabetes] … So how am I gonna know what those were … symptoms for a heart attack … the only thing that was wrong was that my chest was hurting … Very uncomfortable … I didn't know what I was feeling … my heart's always been good.

Mrs. Lorenzo: “just having chest pains and back pains and I thought it was just a cold … or possibly, pneumonia, like I had twenty years ago … I wasn't feeling, you know like … when somebody works or something you know, you can feel tired coming from work and everything but I didn't … I just felt like I was run down, tired and I don't know if it was because my blood sugar was high or my, you know anemia, or whatever it was.” She “never gave it a second thought … because I never considered myself that sick … being that I had a heart attack before you know … that never dawned on me that way.”

## Discussion

This is the first multicenter study utilizing prospective patient recruitment analyzing in-depth initial and follow-up interviews in conjunction with medical record reviews to create a typology of AMI treatment-seeking behavior for Hispanic and non-Hispanic women. It provides insights into the genesis of the delay phenomena commonly observed among ethnic minority women experiencing AMI. We found that Hispanic women were significantly more likely to delay care for two major reasons: (1) misinterpretation of AMI symptoms with failure to take appropriate action, and (2) misinterpretation of symptoms by medical providers leading to delayed treatment.

Few studies have focused on the relationship between women's perception of symptomatology and treatment-seeking behavior during AMI. Various typologies have been proposed by authors attempting to stratify women by treatment-seeking behavior.^4,7,12–14^ Most of these typologies have been created based on single-session interviews of women who have recently experienced an AMI. Davis et al. utilized grounded theory to analyze interviews with nine women who sought care for acute coronary syndrome at a single institution; the authors divided women into those who did and did not immediately recognize their symptoms.^13^ Both Arslanian-Engoren et al. and Gyberg et al. conducted qualitative studies of <20 women with AMI and described symptom variation and patient uncertainty/hesitance as factors negatively influencing treatment-seeking behavior.^12,14^ Lichtman et al. performed a qualitative study of 30 women 30–55 years of age retrospectively identified after hospitalization with AMI. Five themes were identified in this article: prodromal variation, inaccurate self-identification of risk, competing/conflicting priorities, lack of recognition by health care providers, and limited historical utilization of primary care services.^7^

Delayed AMI presentations resulting from participants' misinterpretation of symptoms continue to challenge public health educators and primary care and emergency medicine providers, despite public education efforts targeting symptom awareness.^8,13,26^ We sought to expand current understanding of this problem by identifying a cohesive typology to explore psychosocial dimensions affecting the decision to seek care for AMI symptoms. Our typology stratified four primary categories of behavior based on delay time, ischemic symptom recognition, and actions that elucidate differences between short and long delayers.

Fast Actors correctly perceived themselves as highly susceptible to AMI and their symptoms as serious. In keeping with current American Heart Association public recommendations, these women immediately sought emergency services either through calling 911 or presenting to an emergency department.^27^ They appropriately believed that the potential benefits of getting help outweighed possible barriers such as financial burdens and/or lack of health insurance. Got Luckies received prompt treatment due to the intervention of friends or family members. These two women failed to perceive their symptoms as ischemic, yet were willing to seek care upon insistence from a trusted or influential person in their lives.

By contrast, The Derailed initially sensed the need for prompt medical care but minimized symptom importance after health care providers suggested their symptoms were noncardiac in origin. Health care provider response to these women's concerns before the index hospitalization unfavorably affected their behavior, contributing to extended prehospital delay time. Experiences of The Derailed women present challenges for improving provider recognition of vague symptoms of cardiac ischemia without creating “malignant attention,”^28^ causing unnecessary alarm, and excessive health care expenditures. Furthermore, the behavior described by The Derailed type suggests a need for additional research to explore how vague, intermittent symptoms are addressed by health care providers and how the interpretation of these symptoms is communicated to and received by patients, particularly Spanish-speaking women.

Another group with ineffective treatment-seeking behavior is the Wonder a Lots. Beyond failing to recognize their symptoms as ischemic, they over relied on self-treatment in an extended process of assigning various noncardiac causes to their symptoms. This group had the least insight regarding the need to promptly seek care and did not understand the boundary between ending self-care and seeking medical attention. Ironically, several of these women had extensive experience with the health care system due to chronic medical conditions, yet lacked insight into the significance of their AMI symptoms.

### Health equity implications

Our study detailed three key findings regarding treatment-seeking behavior among women experiencing AMI. First, all women included in our sample described a broad range of symptoms and assessment strategies. Hispanic women expressed different symptom assessment, reporting, and care-seeking strategies compared with non-Hispanic women, further emphasizing the need to understand ethnic factors that shape patient perceptions. This ethnic variation suggests that health care providers and laypersons should maintain a higher index of suspicion for cardiac ischemia in Hispanic women. Second, health care system barriers and variable communication with providers contributed to participant delay. Third, in concordance with existing literature,^1,4–9,12–15,29–31^ intermittent ischemic symptoms were common but often underappreciated by women and health care providers. In the context of understanding women's treatment-seeking behavior, providers must assess the presence of prodromal complaints to identify early warning symptoms of impending AMI. Intermittent atypical complaints are ubiquitous in women; clinicians should be encouraged to inquire about potential complaints in the weeks preceding initial presentation to capture these sentinel symptoms among vulnerable patients.

### Limitations

Medical records have inherent limitations relating to completeness. Although our sample is robust for a qualitative study, the small size and convenience sampling strategy limit generalizability and the ability to demonstrate statistically significant differences in clinical outcomes. Our analyses focused solely on women who survived their AMI and may not represent the experiences of those who never sought care or experienced fatal out-of-hospital events. Lastly, recall bias likely impacted women interviewed farther out from their index AMI, although the delay was often medically necessary for unstable participants and those with protracted hospitalizations.

## Conclusions

Women of differing ethnicity manifest variation in AMI symptomatology, perceived symptom severity, and treatment-seeking behavior. Ethnic variations should be considered to minimize barriers confronting long delayers with regard to assessment strategies, response to symptoms, and encounters within the health care system. Treatment-seeking patterns indicate a need for education of women and health care providers to facilitate timely recognition of AMI symptoms. Given the direct correlation between treatment delay time and mortality,^1,2,28,29,32,33^ further understanding of our typology may contribute to improved AMI outcomes in women. Primary care providers have a key role influencing the trajectory of women's treatment-seeking behavior. Future research on tailored educational outreach regarding symptom recognition and appropriate actions that are ethnically informed may reduce health disparities in AMI care among Hispanic women.

## Abbreviations Used

ACS: American Cancer Society
AMI: acute myocardial infarction
HRSA: Health Resources and Services Administration
PCT: Patient Career Timeline

## References

[1] Mehta LS, Beckie TM, DeVon HA, et al. Acute myocardial infarction in women: a scientific statement from the American Heart Association. Circulation. 2016;133:916–947 26811316 10.1161/CIR.0000000000000351

[2] Benjamin EJ, Virani SS, Callaway CW, et al. Heart disease and stroke statistics—2018 update: a report from the American Heart Association. Circulation. 2018;137:e67–e492 29386200 10.1161/CIR.0000000000000558

[3] Bullock-Palmer RP Prevention, detection and management of coronary artery disease in minority females. Ethn Dis. 2015;25:499 26674268 10.18865/ed.25.4.499 PMC4671445

[4] Harralson TL Factors influencing delay in seeking treatment for acute ischemic symptoms among lower income, urban women. Heart Lung. 2007;36:96–104 17362790 10.1016/j.hrtlng.2006.08.002

[5] Loboz-Grudzień K, Jaroch J Women with acute coronary syndromes have a worse prognosis—why? The need to reduce “treatment-seeking delay.” Cardiol J. 2011;18:219–221 21660910

[6] Franceski BD Cardiovascular health in women: an overview of gender-related issues. Adv Emerg Nurs J. 2009;31:63–72 20118855 10.1097/TME.0b013e318195ef15

[7] Lichtman JH, Leifheit-Limson EC, Watanabe E, et al. Symptom recognition and healthcare experiences of young women with acute myocardial infarction. Circ Cardiovasc Qual Outcomes. 2015;8:S31–S38 25714826 10.1161/CIRCOUTCOMES.114.001612 PMC4801001

[8] Foa C, Fuochi G, Fruggeri L Factors affecting women's well-being during the experience of acute myocardial infarction: a literature review. Acta Biomed Ateneo Parmense. 2015;86:51–61 25835766

[9] Gupta A, Wang Y, Spertus JA, et al. Trends in acute myocardial infarction in young patients and differences by sex and race, 2001 to 2010. J Am Coll Cardiol. 2014;64:337–345 25060366 10.1016/j.jacc.2014.04.054 PMC4415523

[10] McSweeney JC, Crane PB Challenging the rules: women's prodromal and acute symptoms of myocardial infarction. Res Nurs Health. 2000;23:135–146 10782872 10.1002/(sici)1098-240x(200004)23:2<135::aid-nur6>3.0.co;2-1

[11] Schoenberg NE, Peters JC, Drew EM Unraveling the mysteries of timing: women's perceptions about time to treatment for cardiac symptoms. Soc Sci Med. 2003;56:271–284 12473313 10.1016/s0277-9536(02)00026-6

[12] Arslanian-Engoren C, Scott LD Delays in treatment-seeking decisions among women with myocardial infarction. Dimens Crit Care Nurs. 2017;36:298–303 28777117 10.1097/DCC.0000000000000260

[13] Davis LL, Mishel M, Moser DK, et al. Thoughts and behaviors of women with symptoms of acute coronary syndrome. Heart Lung. 2013;42:428–435 24011604 10.1016/j.hrtlng.2013.08.001 PMC3818316

[14] Gyberg A, Björck L, Nielsen S, et al. Women's help-seeking behaviour during a first acute myocardial infarction. Scand J Caring Sci. 2016;30:670–677 26582252 10.1111/scs.12286

[15] Arslanian-Engoren C Black, Hispanic, and white women's perception of heart disease. Prog Cardiovasc Nurs. 2007;22:13–19 17342001 10.1111/j.0889-7204.2007.05698.x

[16] Dracup K, Moser DK, Eisenberg M, et al. Causes of delay in seeking treatment for heart attack symptoms. Soc Sci Med. 1995;40:379–392 7899950 10.1016/0277-9536(94)00278-2

[17] Lee HO, Bahler R, Taylor A, et al. Clinical symptoms of myocardial infarction and delayed treatment-seeking behavior in blacks and whites. J Appl Biobehav Res. 1998;3:135–159

[18] Carpenter CJ A meta-analysis of the effectiveness of health belief model variables in predicting behavior. Health Commun. 2010;25:661–669 21153982 10.1080/10410236.2010.521906

[19] Strecher VJ, Rosenstock IM Chapter 3: the health belief model. In: Health Behavior and Health Education: Theory, Research and Practice, 2nd ed. Edited by Glanz K, Lewis FM, Rimer BK San Francisco, CA: Jossey-Bass Publishers, 1997, pp. 41–59

[20] Leventhal H, Cameron L Behavioral theories and the problem of compliance. Patient Educ Couns. 1987;10:117–138

[21] Dempsey SJ, Dracup K, Moser DK Women's decision to seek care for symptoms of acute myocardial infarction. Heart Lung. 1995;24:444–456 8582820 10.1016/s0147-9563(95)80022-0

[22] U.S. Census Bureau. Quick facts: Colorado. Available at www.census.gov/quickfacts/CO Accessed 44, 2018

[23] Thygesen K, Alpert JS, Jaffe AS, et al. Third universal definition of myocardial infarction. Eur Heart J. 2012;33:2551–2567 22922414 10.1093/eurheartj/ehs184

[24] Olson JD, McAllister C, Grinnell LD, et al. Applying constant comparative method with multiple investigators and inter-coder reliability. Qual Rep. 2016;21:26

[25] Kleinman A Chapter 2: the personal and social meaning of illness. In: *The Illness Narratives: Suffering, Healing, and the Human Condition.* Edited by The Free Press, a Division of Simon & Schuster. New York: Basic Books, Inc., 1988, pp. 48–32

[26] Henriksson C, Larsson M, Arnetz J, et al. Knowledge about acute myocardial infarction (AMI) and attitudes to medical care seeking-a comparison between patients and the general public. Open J Nurs. 2012;2:372–378

[27] American Heart Association. Warning signs of a heart attack. January 11, 2018. Available at www.heart.org/HEARTORG/Conditions/HeartAttack/WarningSignsofaHeartAttack/Warning-Signs-of-a-Heart-Attack_UCM_002039_Article.jsp#.WujPTMgh2L0 Accessed: May 1, 2018

[28] Alonzo AA The impact of the family and lay others on care-seeking during life-threatening episodes of suspected coronary artery disease. Soc Sci Med. 1986;22:1297–1311 3738555 10.1016/0277-9536(86)90093-6

[29] Clark LT, Bellam SV, Shah AH Analysis of prehospital delay among inner-city patients with symptoms of myocardial infarction: implications for therapeutic intervention. J Nat Med Assoc. 1992;84:931–937 PMC2571733 1460679

[30] Bahr RD Failure to recognize prodromal symptoms in patients with acute myocardial infarction and missing out on a way to reduce time to treatment. Am J Cardiol. 2002;90:446–447 10.1016/s0002-9149(02)02543-2 12161245

[31] Hofgren C, Karlson BW, Herlitz J Prodromal symptoms in subsets of patients hospitalized for suspected acute myocardial infarction. Heart Lung. 1995;24:3–10 7706097 10.1016/s0147-9563(05)80089-5

[32] Neubeck L, Maiorana A Time to get help? Acute myocardial infarction and delay in calling an ambulance. Heart Lung Circ. 2015;24:1–3 25201029 10.1016/j.hlc.2014.08.005

[33] Mnatzaganian G, Braitberg G, Hiller JE, et al. Sex differences in in-hospital mortality following a first acute myocardial infarction: symptomatology, delayed presentation, and hospital setting. BMC Cardiovasc Disord. 2016;16:109 27389522 10.1186/s12872-016-0276-5 PMC4937590

